# Interaction of Staphylococci with Human B cells

**DOI:** 10.1371/journal.pone.0164410

**Published:** 2016-10-06

**Authors:** Tyler K. Nygaard, Scott D. Kobayashi, Brett Freedman, Adeline R. Porter, Jovanka M. Voyich, Michael Otto, Olaf Schneewind, Frank R. DeLeo

**Affiliations:** 1 Laboratory of Bacteriology, Rocky Mountain Laboratories, National Institute of Allergy and Infectious Diseases, National Institutes of Health, Hamilton, Montana, United States of America; 2 Department of Microbiology and Immunology, Montana State University, Bozeman, Montana, United States of America; 3 Laboratory of Bacteriology, National Institute of Allergy and Infectious Diseases, National Institutes of Health, Bethesda, Maryland, United States of America; 4 Department of Microbiology, University of Chicago, Chicago, Illinois, United States of America; Institut Pasteur, FRANCE

## Abstract

*Staphylococcus aureus* is a leading cause of human infections worldwide. The pathogen produces numerous molecules that can interfere with recognition and binding by host innate immune cells, an initial step required for the ingestion and subsequent destruction of microbes by phagocytes. To better understand the interaction of this pathogen with human immune cells, we compared the association of *S*. *aureus* and *S*. *epidermidis* with leukocytes in human blood. We found that a significantly greater proportion of B cells associated with *S*. *epidermidis* relative to *S*. *aureus*. Complement components and complement receptors were important for the binding of B cells with *S*. *epidermidis*. Experiments using staphylococci inactivated by ultraviolet radiation and *S*. *aureus* isogenic deletion mutants indicated that *S*. *aureus* secretes molecules regulated by the SaeR/S two-component system that interfere with the ability of human B cells to bind this bacterium. We hypothesize that the relative inability of B cells to bind *S*. *aureus* contributes to the microbe’s success as a human pathogen.

## Introduction

*Staphylococcus aureus* is a ubiquitous Gram-positive bacterium capable of causing life-threatening disease in humans and animals alike. This prominent pathogen has remained a major cause of morbidity and mortality despite the advent of antibiotic therapy [1]. For example, there are an estimated 72,444 cases of invasive MRSA infection and 9,194 associated patient deaths in the United States annually [2]. The need to advance novel therapeutic strategies to successfully treat *S*. *aureus* infection is further underscored by the high burden of community-associated MRSA (CA-MRSA) in the US [3]. Distinct CA-MRSA lineages appear to have obtained beta-lactam resistance via horizontal acquisition of *mecA* on multiple separate occasions outside of the healthcare setting [4], and are noted for their enhanced virulence relative to some of the most successful healthcare-associated MRSA (HA-MRSA) lineages. Despite extensive efforts for more than 100 years [5], attempts to vaccinate humans against *S*. *aureus* infections have repeatedly failed in clinical trials [6].

The ability of *S*. *aureus* to cause disease is largely attributed to the expression of an extensive and often redundant array of virulence genes that includes various toxins, adhesins, and immunomodulatory proteins. In addition, numerous *S*. *aureus* virulence molecules are believed to interfere with binding and subsequent phagocytosis of invading bacteria by host immune cells. The expression of these virulence molecules *in vivo* is largely coordinated by the concerted influence of two-component sensory systems, 16 of which have been putatively identified by sequence analysis within the *S*. *aureus* genome [7]. Of these two-component systems, the accessory gene regulator (Agr) and the regulator of *S*. *aureus* exoprotein expression (SaeR/S) are perhaps the best studied. Many *S*. *aureus* virulence genes, and extracellular toxins in particular, are under regulation by Agr and/or SaeR/S.

The ingestion of bacteria by phagocytes is primarily initiated by the engagement of type I Fc receptors and complement receptors with corresponding opsonins on the bacterial surface. *S*. *aureus* expresses two proteins, *S*. *aureus* protein A (Spa) and immunoglobulin G-binding protein (Sbi), that can inhibit binding of *S*. *aureus* specific IgG with Fc receptors [8, 9]. A number of other secreted proteins, including staphylococcal complement inhibitor (SCIN) and extracellular fibrinogen-binding protein (Efb), have the capacity to inhibit the complement pathway to prevent deposition of activated C3 and C4 derivatives on the bacterial surface [10]. Compared to the closely related and common skin commensal *Staphylococcus epidermidis*, the large number of proteins expressed by *S*. *aureus* that have the ability to interfere with Fc and complement receptor binding suggests that blocking the action of these receptors is an important component of *S*. *aureus* fitness. An extensive body of research has elucidated the activity of these proteins *in vitro* and examined their importance in animal models of *S*. *aureus* infection. However, much less is known about the concerted influence of these immune evasion molecules on *S*. *aureus* pathogenesis during human infection.

To better understand the influence of *S*. *aureus* virulence molecules on the recognition of bacteria by human immune cells, we investigated the interaction of *S*. *aureus* and *S*. *epidermidis* with leukocytes in human blood *ex vivo*. These studies revealed a significant difference in the association of *S*. *aureus* with human B cells relative to *S*. *epidermidis* or zymosan. Inactivation of *S*. *aureus* with ultraviolet irradiation promoted its association with human B cells, while an *S*. *aureus* mutant lacking genes encoding SaeR/S bound to human B cells in a manner seemingly indistinguishable from that of *S*. *epidermidis*. The association of B cells with *S*. *epidermidis* was found to be largely mediated by complement receptor 2 (CR2) and activated complement components. Collectively, these findings suggest that one or more molecules secreted by *S*. *aureus* limit the ability of human B cells to associate with this pathogen.

## Materials and Methods

### Human Subjects Research

These studies were approved by the Institutional Review Board for Human Subjects, US National Institute of Allergy and Infectious Diseases, National Institutes of Health (protocol number 01-I-N055). Studies were conducted according to the policies provided in the Declaration of Helsinki. Each volunteer gave written informed consent prior to participation in the study.

### Bacteria Strains and Culture Conditions

*S*. *aureus* and *S*. *epidermidis* strains were cultured in tryptic soy broth (TSB; EMD Millipore) in a rotary shaker at 225 rpm and 37°C. USA400 isogenic *saeR/S* (USA400Δ*saeR/S*) [11] and *agr* (USA400Δ*agr*) [12] deletion strains, and the Newman isogenic *spa* deletion strain (NewmanΔ*spa*) [13] were generated in previous studies. Pulsed-field type USA100, USA200 (EMRSA16), and USA300 (LAC) strains have been described previously [14–16]. For all assays, overnight bacteria cultures were used to inoculate 20 mL TSB (1:100 dilution). Cultures were grown to mid-exponential growth phase (ME; defined by OD_600_ = 0.75 using a Molecular Devices SpectraMax Plus 384), harvested by centrifugation (5,000 × *g*, 5 min, 4°C), and washed with DPBS. For flow cytometry experiments, staphylococci (2 × 10^8^ CFU) or zymosan (2 × 10^8^ particles) were resuspended with 10 μg/mL fluorescein-5-isothiocyanate (FITC; ThermoFisher Scientific) in 1 mL of Dulbecco’s phosphate buffered saline (DPBS; Sigma-Aldrich) and incubated for 15 min at 37°C with agitation at 1400 rpm every 30 seconds using an Eppendorf Thermomixer R. Following FITC-labeling, samples were washed and resuspended in DPBS. For inactivation of bacteria by ultraviolet (UV) irradiation, 1 × 10^9^ CFU of FITC-labeled bacteria in 45 mL DPBS were UV irradiated for 30 min using a UVC 500 ultraviolet crosslinker (Hoefer, Inc). Following UV treatment, samples were centrifuged (3,200 × *g*, 4°C, 5 min) and resuspended in DPBS. Bacteria concentrations were determined by plating diluted samples on tryptic soy agar (EMD Millipore).

### Flow cytometry analysis of *ex vivo* human blood infection

Infection of human blood *ex vivo* was performed as previously described [17] using FITC-labeled bacteria. Briefly, indicated concentrations of FITC-labeled bacteria in 100 μL of DPBS were combined with 1 mL of freshly drawn human blood containing heparin (Fresenius Kabi; 20 USP units/mL) in 2.0-mL microcentrifuge tubes and incubated at 37°C with end-over-end rotation. Preliminary experiments in which sodium citrate was used as an anticoagulant yielded results similar to those with heparinized blood. At indicated times, red blood cells were lysed using erythrocyte lysis buffer EL (Qiagen) following the manufacturer’s protocol (QIAamp RNA Blood Mini-Handbook), and leukocytes were resuspended in 200 μL ice cold DPBS. Pelleted cells were divided into 50-μL aliquots, stained with APC Mouse Anti-Human CD3, APC Mouse Anti-Human CD14, APC Mouse Anti-Human CD19, or PE Mouse Anti-Human CD66c (BD Biosciences), and analyzed using flow cytometry (FACSCalibur, BD Biosciences). Forward and side-scatter were used to set gates on leukocyte populations (lymphocytes, monocytes, and granulocytes). B cells were defined as CD19^+^ leukocytes, T cells as CD3^+^ leukocytes, monocytes as CD14^+^ leukocytes, and PMNs as leukocytes expressing high levels of surface CD66 (CD66^High^).

### Immunofluorescence microscopy analysis of isolated B cells and monocytes

Unlabeled bacteria in 200 μL of DPBS were combined with 4 mL of freshly drawn heparinized human blood in 5-mL polypropylene culture tubes and incubated at 37°C with end-over-end rotation. At indicated times, red blood cells were lysed as described above and negative isolation of human B cells and monocytes was performed using Dynabeads Untouched Human B cell Kit or Dynabeads Untouched Human Monocyte Kit (Life Technologies), respectively, following the manufacturer’s protocol. Purified cells were deposited onto poly-L-lysine (Sigma-Aldrich) coated slides by centrifuging at 300 rpm for 3 min using a cytocentrifuge (Thermo Scientific Shandon Cytospin 4). Intracellular and extracellular staining of bacteria was then performed as previously described [18]. Differentially stained slides were used for immunofluorescence microscopy (Zeiss Axioskop 2 Plus coupled with an Olympus DP73 color camera).

### *In vitro* assays with human PBMCs and staphylococci

Peripheral blood mononuclear cells (PBMCs) and corresponding normal human serum (NHS) were isolated from venous blood of healthy individuals. Human PBMCs were combined with FITC-labeled bacteria *in vitro* as previously described [19]. For isolation of PBMCs, heparinized blood was incubated for 20 min at room temperature at a 1:1 ratio with 0.9% sodium chloride (Injection USP; Baxter Healthcare) containing 3.0% Dextran (Sigma-Aldrich) to sediment erythrocytes. The leukocyte-containing supernatant was centrifuged at 825 × *g* for 10 min and resuspended in 35 mL of 0.9% sodium chloride. The cell suspension was underlaid with 10 mL of Ficoll-Paque PLUS (1.077 g/L; GE Healthcare) and centrifuged at 448 × *g* for 17 min to separate PBMCs. PBMCs in the Buffy coat were aspirated, washed with DPBS, and enumerated using a hemocytometer. PBMCs were then resuspended in RPMI 1640 medium (Life Technologies) buffered with 10 mM HEPES (RPMI/H, pH 7.2) to a final concentration of 1 × 10^7^ cells/mL. Purified human PBMCs (1 × 10^6^ cells) were then combined with opsonized FITC-labeled *S*. *epidermidis* (1 × 10^6^ CFUs) or *S*. *aureus* (1 × 10^6^ CFUs) in 1 mL RPMI/H and incubated at 37°C with end-over-end rotation. At indicated times, samples were stained with APC-coupled mouse anti-human CD19 mAb and analyzed using flow cytometry as described above.

For complement inhibition assays, 1 mL of fresh NHS diluted by 1:10 with DPBS was heat-treated by incubating at 56°C for 30 min with intermittent shaking at 1400 rpm. Complement was depleted from NHS by incubating 100 μL of NHS with 20 μg of cobra venom factor (CVF; Sigma-Aldrich) at 37°C for 60 min with intermittent shaking at 1400 rpm. Samples were then diluted 1:10 with DPBS. Complement activation in NHS was inhibited by combining 100 μL of NHS with 50 μg of FUT-175 (BD Biosciences), and the treated NHS was then diluted 1:10 with DPBS. FITC-labeled *S*. *epidermidis* strain 1456 (1 mL; 2 × 10^8^ CFU/mL) was centrifuged (5,000 × *g*, 5 min, 4°C) and resuspended in 10% NHS, NHS treated with CVF, NHS treated with FUT-175, heat-treated NHS, or DPBS. Samples were then incubated at 37°C for 15 min with intermittent shaking at 1400 rpm. NHS-opsonized bacteria (or those treated with DPBS) were then washed with DPBS and resuspended in RPMI/H to a final concentration of 2 × 10^8^ CFU/mL.

For assays that used antibodies specific for complement receptors, purified human PBMCs were combined with 5 μg of mouse anti-human CD21, CD35, CD11b, or CD11c mAbs (BD Biosciences) immediately prior to addition of FITC-labeled *S*. *epidermidis* (1 × 10^6^ CFUs) or FITC-labeled USA400Δ*saeR/S* (1 × 10^6^ CFUs) opsonized with NHS. For assays that used recombinant complement receptors, 1 × 10^6^ CFUs of opsonized FITC-labeled *S*. *epidermidis* were combined with 4 μg of recombinant CD21, CD35, CD11b, or CD11c (R&D Systems) and incubated on ice for 10 min prior to being added to purified human PBMCs.

## Results

### A higher proportion of B cells associate with *S*. *epidermidis* compared to *S*. *aureus*

**Fig 1:**
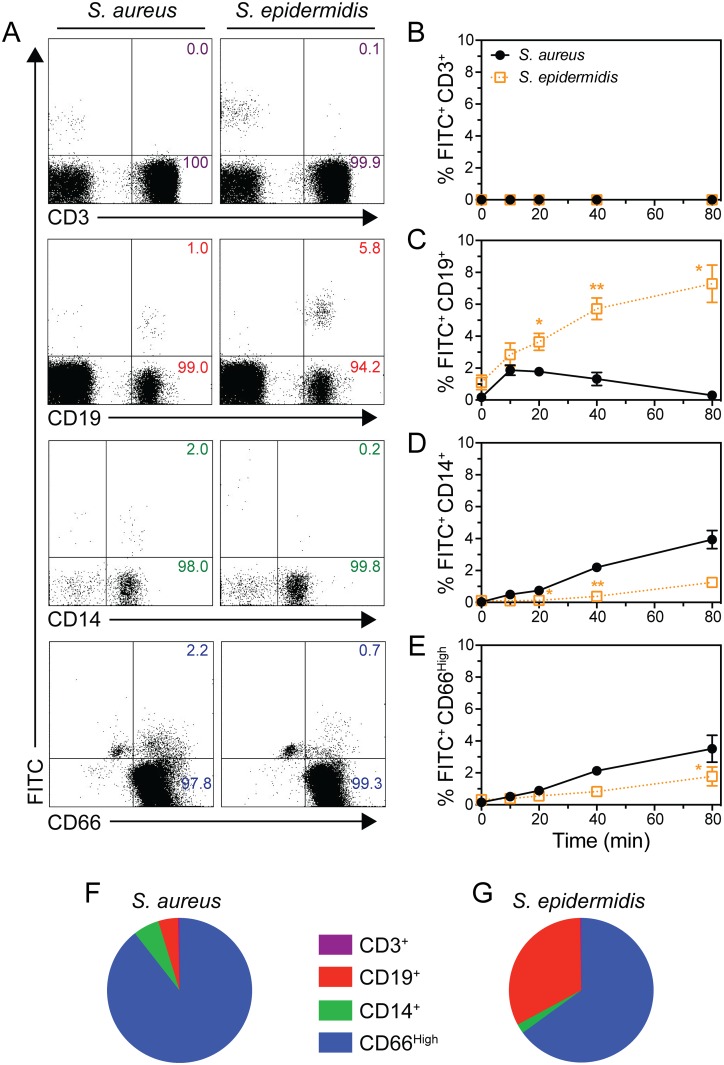
Association of *S*. *aureus* or *S*. *epidermidis* with PMNs, monocytes, B cells, and T cells in human blood. **A**) Representative flow cytometry dot plots of CD3^+^ lymphocytes (T cells), CD19^+^ lymphocytes (B cells), CD14^+^ monocytes, and CD66^High^ granulocytes (PMNs) 40 min after combining heparinized human blood with FITC-labeled *S*. *aureus* or *S*. *epidermidis*. **B-E**) Flow cytometry analyses of leukocyte subsets associated with FITC-labeled *S*. *aureus* or *S*. *epidermidis*. **F & G**) Relative distribution of CD66^High^ granulocytes, CD14^+^ monocytes, CD19^+^ lymphocytes, and CD3^+^ lymphocytes associated with *S*. *aureus* or *S*. *epidermidis* at 40 min. Data are from experiments presented in panels B-E. Data in all panels represent 3 experiments with different blood donors using 5 × 10^5^ CFU/mL bacteria. *P<0.05 and **P<0.01 versus *S*. *aureus* as determined with a paired two-tailed Student’s t test.

*S*. *aureus* expresses a number of cell surface and freely secreted proteins that have capacity to inhibit the ability of phagocytes, such as polymorphonuclear leukocytes (PMNs) and monocytes, to bind and internalize this pathogen. To identify differences in the binding of human immune cells with *S*. *aureus* relative to other bacteria, we used flow cytometry to compare the association of PMNs, monocytes, B cells, and T cells with CA-MRSA strain USA300 (USA300) or *S*. *epidermidis* strain 1457 in heparinized human blood (Fig 1). Unexpectedly, there was a time-dependent increase in the number of B cells (CD19^+^ lymphocytes) in human blood that were associated with *S*. *epidermidis* (Fig 1C). By comparison, there were significantly fewer B cells associated with *S*. *aureus*. Using these assay conditions (a relatively low bacteria-to-PMN ratio), a higher proportion of blood monocytes (CD14^+^ monocytes) and PMNs (CD66^High^ granulocytes) were associated with *S*. *aureus* compared to *S*. *epidermidis*, especially at late time points (Fig 1D and 1E). However, the apparent differences in PMN association between *S*. *aureus* and *S*. *epidermidis* can largely be attributed to rapid destruction of *S*. *epidermidis* after phagocytosis [20, 21]. The association of either bacterium with T cells (CD3^+^ lymphocytes) was minimal (Fig 1B). Although the majority of staphylococci were associated with PMNs (Fig 1F and 1G, blue shading), more than 30% of *S*. *epidermidis* positive cells were B cells (Fig 1G) compared to less than 5% of *S*. *aureus* positive cells (Fig 1F). These findings suggest staphylococci directly associate with B cells in human blood and this interaction is significantly more pronounced for *S*. *epidermidis* relative to *S*. *aureus*.

### Staphylococci are primarily bound to the surface of B cells

**Fig 2:**
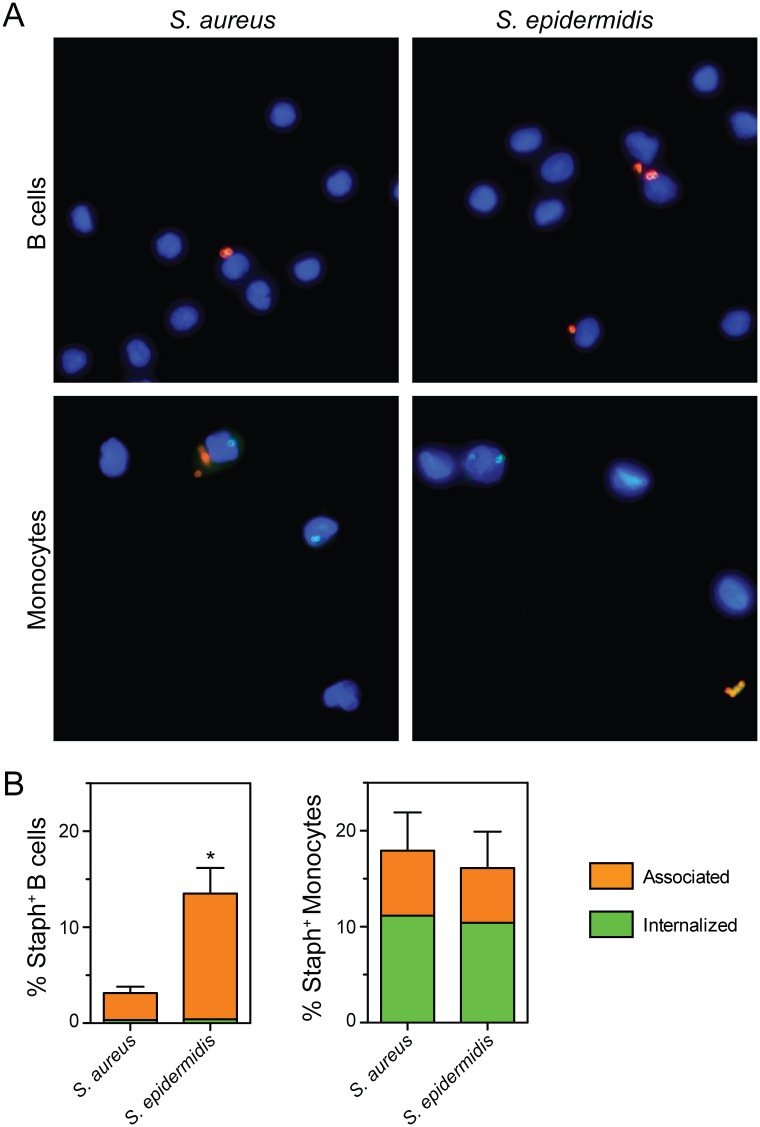
Immunofluorescence microscopy analysis of purified B cells and monocytes associated with *S*. *aureus* or *S*. *epidermidis*. **A**) Representative images (100×) of B cells or monocytes purified from heparinized human blood 80 min after addition of 1 × 10^6^ CFU/mL *S*. *aureus* or *S*. *epidermidis*. Extracellular bacteria are red and internalized bacteria are green (appear turquoise in the context of blue nuclei). **B**) Quantitation of the association and internalization of *S*. *aureus* or *S*. *epidermidis* by B cells or monocytes for assays described in panel A. Data in panel B are the mean ± SEM of at least 5 separate experiments. *P<0.05 for comparison of *S*. *aureus* with *S*. *epidermidis* as determined by paired two-tailed Student’s t test.

Mammalian B cells were recently reported to internalize and kill bacteria [22, 23]. To determine if staphylococci are internalized by B cells in human blood, we used immunofluorescence microscopy to assess binding and ingestion of *S*. *epidermidis* strain 1457 or *S*. *aureus* strain USA300 (Fig 2). In support of the flow cytometry data, a significantly higher proportion of B cells in blood were associated with *S*. *epidermidis* relative to *S*. *aureus*. Both *S*. *epidermidis* and *S*. *aureus* were almost exclusively bound to the surface of B cells and not internalized (Fig 2A and 2B). By comparison, staphylococci associated with monocytes were mostly ingested. These findings corroborate our flow cytometry results and indicate that staphylococci are not ingested by human B cells under the conditions tested.

### B cells associate with zymosan

**Fig 3:**
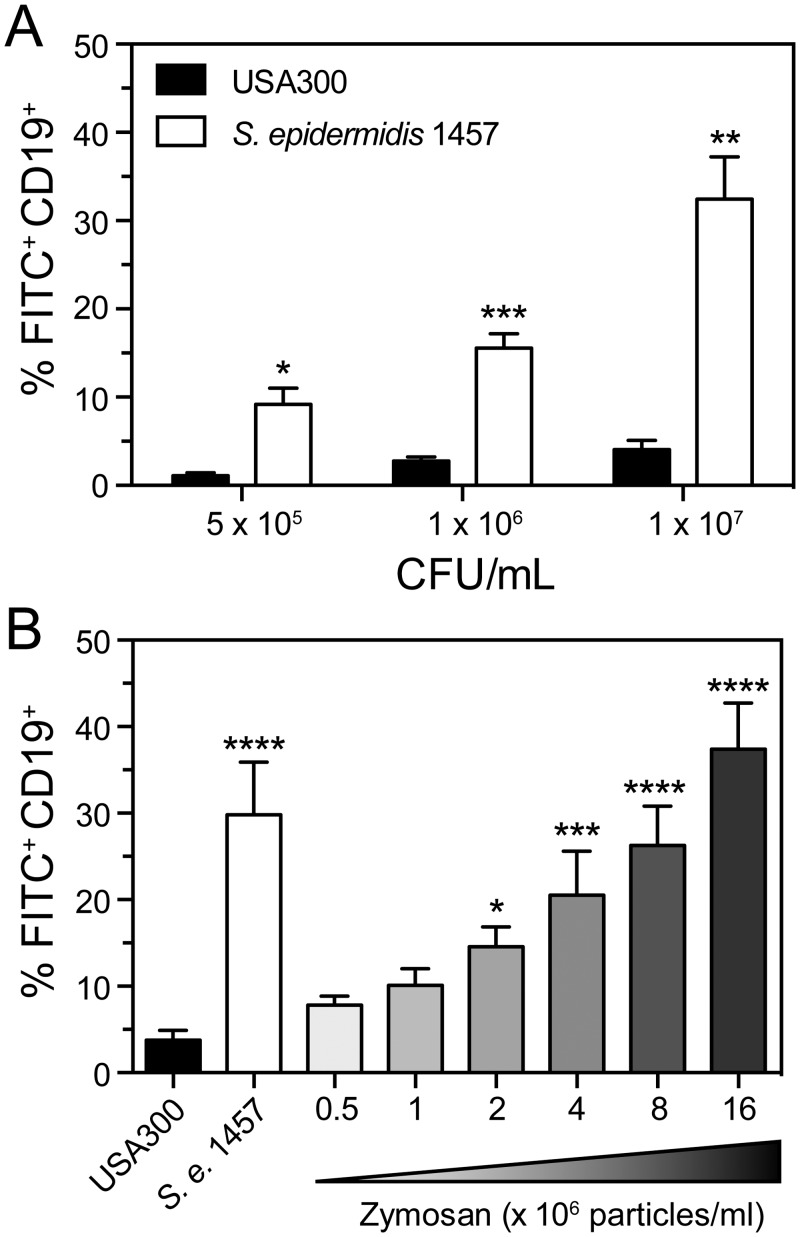
Comparison of the binding of *S*. *aureus*, *S*. *epidermidis* and zymosan with human B cells. **A)** Flow cytometry analysis of B cells associated with *S*. *aureus* strain USA300 or *S*. *epidermidis* strain 1457 in heparinized human blood 40 min after the addition of 5 × 10^5^, 1 × 10^6^, or 1 × 10^7^ CFU/mL of staphylococci. **B)** Percentage of B cells with bound bacteria or zymosan 40 min after combining heparinized human blood with 8 × 10^6^ CFU/mL of FITC-labeled USA300 or *S*. *epidermidis* strain 1457, or the indicated numbers of FITC-labeled zymosan particles. Data in panels A and B are the mean ± SEM of at least 4 separate experiments using different blood donors. *P<0.05, **P<0.01, ***P<0.001, and ****P<0.0001 versus USA300 as determined by paired two-tailed Student’s t test for data in panel A or by repeated-measures one-way ANOVA and Dunnett’s post-test for data in panel B.

Flow cytometry and immunofluorescence microscopy analyses demonstrated that significantly more B cells in blood were associated with *S*. *epidermidis* relative to *S*. *aureus*. This finding was confirmed using different doses (colony-forming units) of bacteria (Fig 3A). The association of *S*. *epidermidis* with the surface of B cells is consistent with the known ability of B cells to directly associate with immune complexes [24–27]. To determine if B cells associate with serum-opsonized particles in general, zymosan—which is readily opsonized by serum complement in blood—was combined with human blood and the percentage of zymosan-associated B cells was measured by flow cytometry (Fig 3B). There was a concentration-dependent increase in the proportion of zymosan-associated B cells in these assays. Compared with *S*. *aureus*, significantly more B cells were associated with zymosan or *S*. *epidermidis*. These findings indicate that B cells readily associate with serum-opsonized particles in human blood, and suggest that the observed limited association of *S*. *aureus* with B cells is atypical.

### Differential association of *S*. *aureus* and *S*. *epidermidis* with B cells is conserved among diverse strains

**Fig 4:**
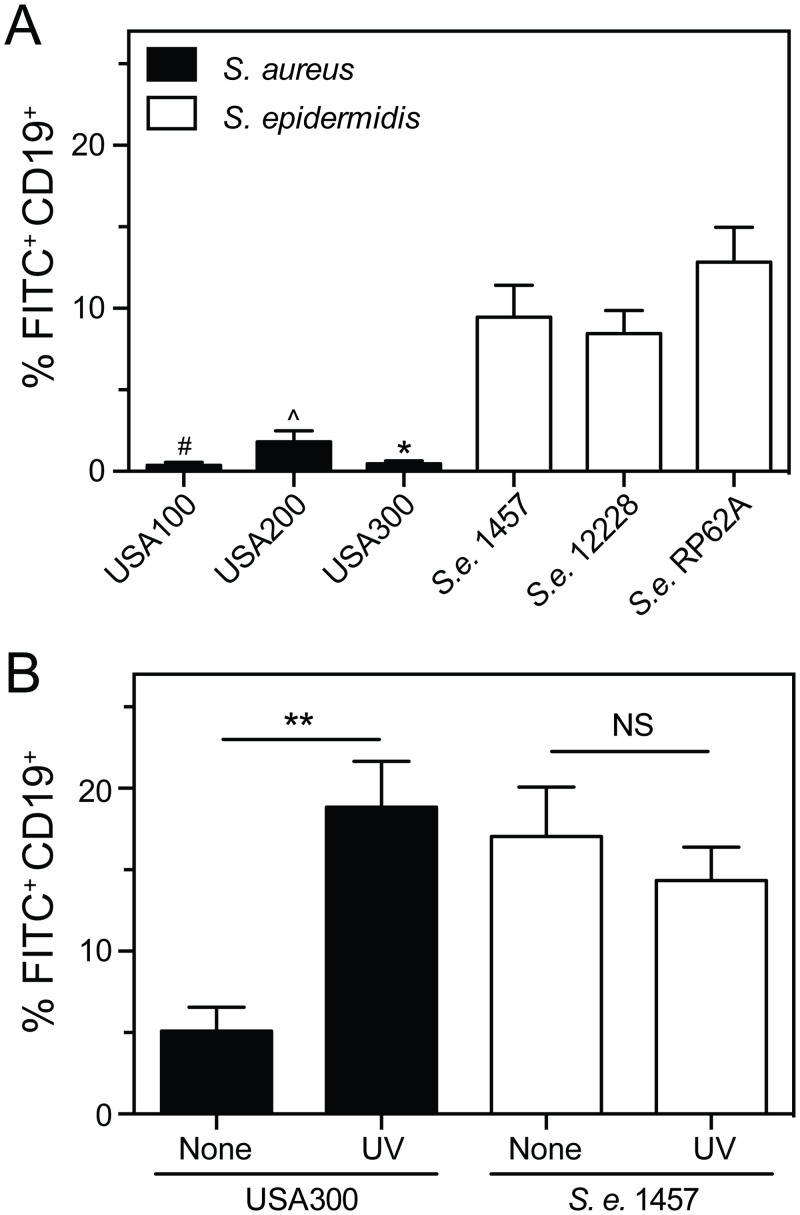
Relative inability of *S*. *aureus* to bind B cells is conserved among diverse strains and influenced by bacterial viability. **A)** Association of B cells with FITC-labeled *S*. *aureus* strains (USA100, USA200, and USA300) or *S*. *epidermidis* strains (1457, 12228, and RP62A) in heparinized human blood was determined by flow cytometry (60 min time point). ~2.5–5 × 10^5^ CFU/mL of bacteria were used in these assays. **B)** Association of B cells with untreated or UV-irradiated USA300 or *S*. *epidermidis* strain 1457 (1 × 10^7^ CFU/mL for each) in heparinized human blood was determined by flow cytometry (40 min time point). Data in panels A and B are the mean ± SEM of 5 separate experiments using different blood donors. For panel A, ^#^, ^, *P≤0.002 versus 1457, 12228, or RP62A as determined by repeated-measures one-way ANOVA and Tukey’s post-test. For panel B, **P<0.01 as determined by paired two-tailed Student’s t test. NS, not significant.

*S*. *aureus* exhibits considerable genetic diversity, with some *S*. *aureus* genome sequences differing by as much as 20% [28]. To determine if the limited association of *S*. *aureus* with B cells is conserved among genetically diverse strains, we used flow cytometry to measure the association of B cells with *S*. *aureus* pulsed-field gel electrophoresis types USA100, USA200, and USA300, as well as *S*. *epidermidis* strains 1457, 12228, and RP62A (Fig 4A). Consistent with our initial findings above, each of the *S*. *epidermidis* strains associated with B cells in human blood, whereas there was similarly limited binding of representative USA100, USA200, or USA300 strains with B cells. These results indicate that the inability of *S*. *aureus* to associate with B cells in human blood is conserved among the diverse *S*. *aureus* strains tested.

### Inactivation of *S*. *aureus* with UV light increases association with B cells

*S*. *aureus* undergoes abrupt changes in gene expression in human blood [29]. These changes include the upregulation of genes encoding numerous secreted toxins and immunomodulatory molecules that are absent in the genome of *S*. *epidermidis*. To determine if molecules secreted by *S*. *aureus* influences its ability to bind B cells, we used ultraviolet (UV) radiation to inactivate USA300 and prevent the active secretion of molecules that potentially block interaction with B cells (Fig 4B). Compared to untreated USA300, there was a significant increase in the percentage of B cells associated with *S*. *aureus* inactivated by UV radiation (Fig 4B). In contrast, UV inactivation of *S*. *epidermidis* had no significant impact on its association with B cells in human blood. These results suggest that molecules secreted by *S*. *aureus* in part inhibit the ability of B cells to bind this bacteria. It is also possible that UV treatment alters bacterial surface molecules—e.g., those that might prevent B cell interaction—such that the binding of *S*. *aureus* with B cells is increased.

### Complement plays a major role mediating the association of *S*. *epidermidis* with B cells

**Fig 5:**
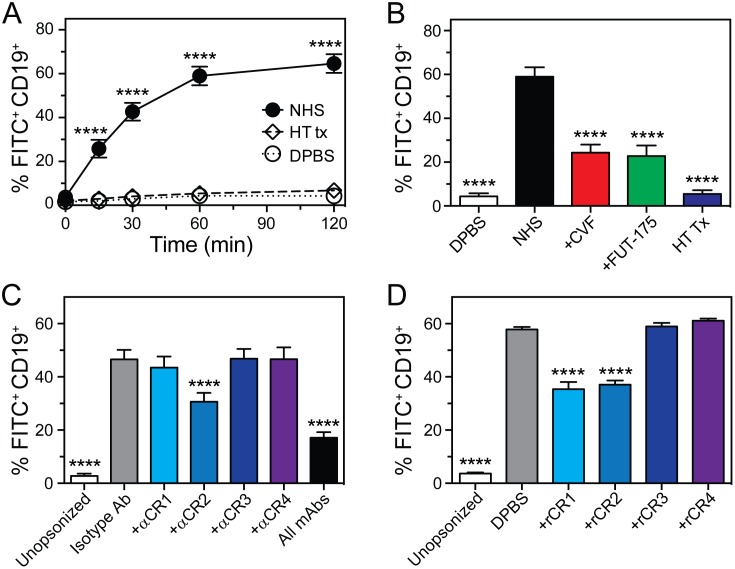
Association of human B cells with *S*. *epidermidis* is mediated primarily by complement. **A)** FITC-labeled *S*. *epidermidis* (strain 1457) was opsonized with NHS, heat-treated NHS (HT Tx), or DPBS (unopsonized control) and binding of B cells to *S*. *epidermidis* was assessed by flow cytometry. **B)** FITC-labeled *S*. *epidermidis* was opsonized with NHS, NHS treated with cobra venom factor (+CVF), NHS treated with FUT-175 (+FUT-175), HT Tx, or DPBS and binding of B cells to *S*. *epidermidis* was assessed by flow cytometry (60 min time point). Data in panels **A** and **B** are the mean ± SEM of 5 separate experiments using different blood donors. **C)** FITC-labeled *S*. *epidermidis* was combined with 5 μg anti-human CD35 (+αCR1), CD21 (+αCR2), CD11b (+αCR3), CD11c (+αCR4), or isotype IgG_2_ control (isotype) mAbs and binding to B cells was determined using flow cytometry (30 min time point). **D)** FITC-labeled *S*. *epidermidis* was combined with 4 μg of soluble recombinant CD35 (+rCR1), CD21 (+rCR2), CD11b (+rCR3), CD11c (+rCR4), or DPBS and binding to B cells was determined using flow cytometry (60 min time point). Data in panels C and D are the mean ± SEM of at least 3 separate experiments using different blood donors. Experiments in panels A-D were performed using 1 × 10^6^ purified human peripheral blood mononuclear cells (PBMCs) and 1 × 10^6^ CFUs *S*. *epidermidis*. B cells were identified as the CD19^+^ leukocyte population of PBMCs. **P<0.01, ***P<0.001, and ****P<0.0001 versus DPBS controls for panels A and D, NHS for panel B, or isotype IgG_2_ for panel C. Statistics were determined by using a repeated-measures one-way ANOVA and Dunnett’s post-test.

Published reports have shown that complement receptor 2 (CR2) expressed by human B cells directly binds to C3d on the antigen surface [24, 30, 31]. As a first step toward determining if complement components are important for the association of *S*. *epidermidis* with B cells, we compared the interaction of B cells with *S*. *epidermidis* that was opsonized with normal human serum (NHS) or heat-inactivated NHS (Fig 5A). Consistent with an involvement of serum complement, there was little or no binding of B cells with *S*. *epidermidis* that had been opsonized with heat-inactivated NHS (Fig 5A). Thus, heat-labile components in human serum are critical for the association of *S*. *epidermidis* with human B cells.

To further determine if complement in NHS influences the association of *S*. *epidermidis* with B cells in our assays, we treated NHS with cobra venom factor (CVF), which depletes complement, or FUT-175 (nafamostat mesilate), a serine protease inhibitor that prevents complement activation, and used the treated NHS in the B cell assays (Fig 5B). Compared with *S*. *epidermidis* opsonized with NHS, there was a significant decrease in the proportion of B cells associated with *S*. *epidermidis* in assays containing NHS that was treated with CVF or FUT-175. These results indicate that complement plays an important role in mediating the association of human B cells with *S*. *epidermidis*.

### Complement receptor 2 is important for the association of *S*. *epidermidis* with human B cells

To determine if one or more complement receptors contribute to the association of *S*. *epidermidis* with B cells, we evaluated the ability of monoclonal antibodies specific for human CD35 (CR1), CD21 (CR2), CD11b (CR3) or CD11c (CR4) to inhibit binding of *S*. *epidermidis* strain 1457 with purified human PBMCs (Fig 5C). Compared to assays with isotype control antibodies, there was a significant decrease in the proportion of B cells associated with *S*. *epidermidis* in assays containing PBMCs pre-treated with anti-CR2 or all complement receptor antibodies combined (Fig 5C). Binding of *S*. *epidermidis* to PBMCs pretreated with all antibodies combined was also significantly less than binding to PBMCs treated with anti-CR2 alone (*P* = 0.0036), suggesting that complement receptors in addition to CR2 play a role in the association of *S*. *epidermidis* with human B cells.

To further investigate the role of individual complement receptors in the association of B cells with staphylococci, we tested the ability of recombinant CD35 (rCR1), CD21 (rCR2), CD11b (rCR3) or CD11c (rCR4) to block association of *S*. *epidermidis* strain 1457 with purified human PBMCs (Fig 5D). Compared to control assays (those lacking recombinant receptors), binding of *S*. *epidermidis* with PBMCs was inhibited significantly by rCR1 or rCR2. Collectively, the antibody blocking data and results with recombinant receptors provide strong support to the idea that CR2 and CR1 contribute to the association of *S*. *epidermidis* with B cells.

### SaeR/S two-component gene regulatory system influences the association of *S*. *aureus* with B cells

**Fig 6:**
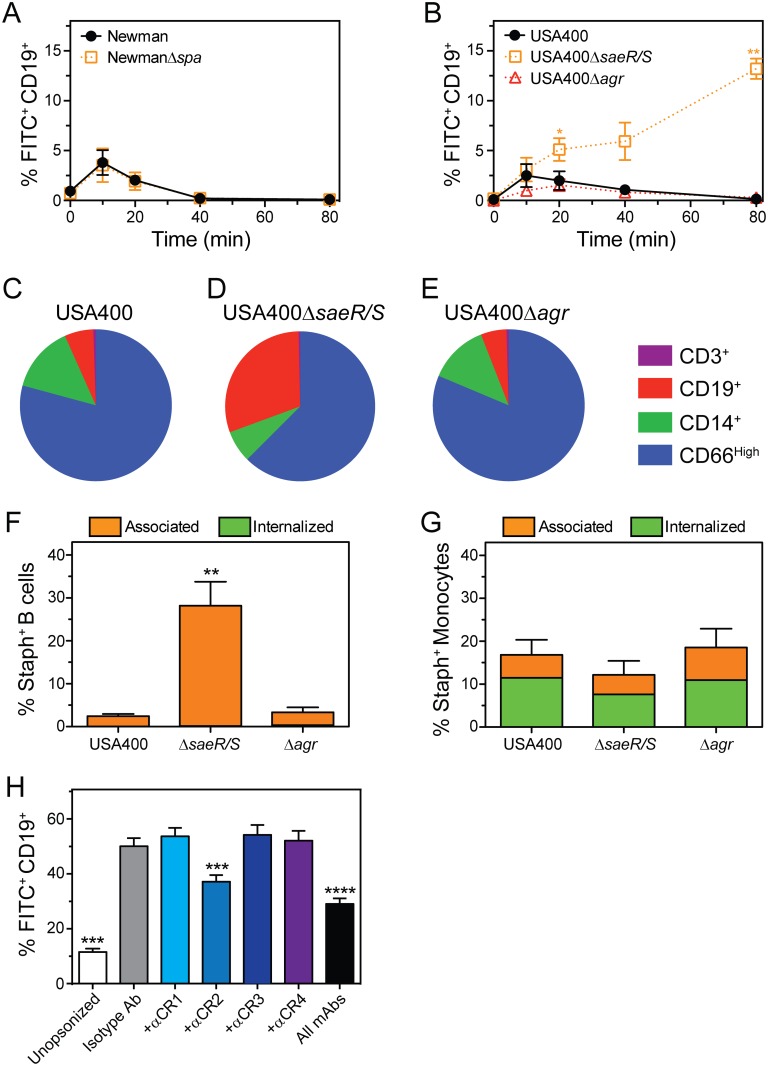
An *S*. *aureus* isogenic *saeR/S* deletion strain associates with human B cells. **A)** FITC-labeled Newman wild-type or isogenic *spa* deletion strains (NewmanΔ*spa*) (each at 5 × 10^5^ CFU/mL) were incubated in heparinized human blood and binding of bacteria to B cells was determined by flow cytometry. **B)** FITC-labeled USA400 wild-type or isogenic *saeR/S* (USA400Δ*saeR/S*) or *agr* (USA400Δ*agr*) deletion strains (each at 5 × 10^5^ CFU/mL) were incubated in heparinized human blood and binding of bacteria to B cells was determined by flow cytometry. Panels A and B are the mean ± SEM of 3 separate experiments using different blood donors. Panels C-E show the relative distribution of CD66^High^ granulocytes, CD14^+^ monocytes, CD19^+^ lymphocytes, and CD3^+^ lymphocytes associated with USA400 **(C)**, USA400Δ*saeR/S* **(D)**, and USA400Δ*agr* **(E)** at 40 min. Data were collected from experiments described in panel B. **F,G)** Association and internalization of USA400, USA400Δ*saeR/S*, or USA400Δ*agr* by purified human B cells **(F)** or monocytes **(G)** was determined 80 min after addition of bacteria (1 × 10^6^ CFU/mL) by using immunofluorescence microscopy. **H)** FITC-labeled USA400Δ*saeR/S* (1 × 10^6^) was combined with 5 μg anti-human CD35 (+αCR1), CD21 (+αCR2), CD11b (+αCR3), CD11c (+αCR4), or isotype IgG_2_ control (Isotype Ab) mAbs and binding to B cells was determined using flow cytometry (30 min time point). Panels F and G are the mean ± SEM of 4 separate experiments using different blood donors. Panel H is the mean ± SEM of 5 separate experiments using different blood donors. Statistics were determined by using a repeated-measures one-way ANOVA and Dunnett’s post-test. For panel F, **P<0.05 versus the wild-type strain (USA400). For panel H, ***P<0.001 and ****P<0.0001 versus isotype IgG_2_.

*S*. *aureus* protein A (Spa) has been shown to inhibit opsonization of bacteria [32] by binding to the Fcγ region of IgG [33] or the Fab domain of V_H_3 clan IgG and IgM [34]. Spa was also reported to have superantigenic properties towards B cells, inducing B cell activation, proliferation, and apoptosis [35]. To determine if Spa influences the association of *S*. *aureus* with B cells in human blood, we compared the binding of Newman wild-type and isogenic *spa*-deletion strains to B cells in human blood using flow cytometry (Fig 6A). In accordance with data using other strains of *S*. *aureus*, there was limited association of wild-type Newman with B cells. These results were mirrored by assays with the *spa* mutant strain (Fig 6A), indicating that Spa does not inhibit association of *S*. *aureus* with human B cells.

The expression of *S*. *aureus* virulence molecules in response to environmental signals is controlled largely by two-component gene regulatory systems, including SaeR/S and accessory gene regulator (Agr). The genes regulated by SaeR/S or Agr only partially overlap [36]. As a step toward identifying the *S*. *aureus* molecules involved in the interaction of this bacterium with human B cells, we compared binding of USA400 wild-type and isogenic *agr*- or *saeR/S* deletion strains (USA400Δ*agr* or USA400Δ*saeR/S*) with B cells and other leukocytes using flow cytometry (Fig 6B–6G). Compared to USA400 wild-type strain, a significantly higher proportion of B cells were associated with USA400Δ*saeR/S*, but not USA400Δ*agr*. The binding of USA400Δ*saeR/S* with B cells was strikingly similar to that of *S*. *epidermidis* (compare data in Figs 1C and 6B), and this includes results with PBMCs pre-treated with anti-CR2 or all complement receptor antibodies combined (Fig 6H; compare Figs 5C and 6H). Compared with monocytes, B cells comprised a larger proportion of USA400Δ*saeR/S* positive cells in human blood (Fig 6C–6E). These findings are consistent with the results using *S*. *epidermidis* (Fig 1G), but contrast with data for the USA400 wild-type or USA400Δ*agr* strains (Fig 6C–6E). For all USA400 strains, the majority of bacteria detected were associated with PMNs, as was observed in our previous experiments (Fig 1F and 1G). We used immunofluorescence microscopy to verify the flow cytometry results and to demonstrate that uptake of bacteria by monocytes was not different between USA400 wild type and isogenic deletion mutant strains (Fig 6F and 6G). Taken together, these results suggest that one or more molecules regulated by SaeR/S inhibit the binding of *S*. *aureus* with B cells in human blood.

## Discussion

Herein we demonstrate that B cells bind *S*. *epidermidis* in human blood. This unexpected finding was not peculiar to *S*. *epidermidis*, since B cells in blood also bound zymosan, a yeast cell wall particle often used for studies of phagocytosis. The association of human B cells with *S*. *epidermidis* was mediated largely by complement and CR2. Previous studies have shown that mammalian B cells bind ICs containing antigen *in vivo* [25–27], and this interaction is mediated by the engagement of CR2 with C3d bound to the antigen surface [24, 30, 31]. In contrast to *S*. *epidermidis*, significantly less *S*. *aureus* was associated with B cells in human blood. The limited association of *S*. *aureus* with B cells was conserved among diverse *S*. *aureus* strains. Notably, inactivation of *S*. *aureus* by UV radiation significantly increased binding of this pathogen to B cells, suggesting one or more molecules actively secreted by *S*. *aureus* (or molecules on the bacterial surface) limit association with B cells. Based on studies with isogenic mutant strains, neither Spa nor molecules regulated by Agr inhibited binding of *S*. *aureus* with B cells. On the other hand, experiments using the USA400Δ*saeR/S* strain indicated that one or more SaeR/S-regulated molecules inhibit the association of *S*. *aureus* with human B cells.

SaeR/S positively regulates the transcription of genes encoding proteins that are known to interfere with the complement pathway [11, 37], including SCIN, extracellular adherence protein (Eap), Sbi, Efb, and extracellular complement binding protein (Ecp). SCIN and Eap have both been shown to bind and stabilize human C3 convertase and prevent deposition of C3b on the surface of *S*. *aureus* [38, 39]. Alternatively, Sbi and Efb have been shown to recruit human plasminogen to the bacterial cell surface [40]. Plasminogen is converted to plasmin by staphylokinase and then acts to degrade IgG and C3b. Sbi has also been shown to bind human C3 [41]. *S*. *aureus* may deploy this protein as a decoy, diverting C3 away from the bacteria and thereby promoting its futile consumption. Efb and Ecp have been reported to play a role in complement evasion by blocking the interaction of C3d with CR2 [42]. Thus, one or more virulence molecules regulated by the SaeR/S two-component system might interfere with the association of B cells with *S*. *aureus* in human blood.

It is not known whether the limited association of B cells with *S*. *aureus* influences pathogenesis or host immune responses *in vivo*. However, it has been demonstrated previously that the binding of CR2 expressed by B cells with C3d on the antigen surface plays several roles that are critical for mounting an effective antibody response. For cognate (antigen-specific) B cells, co-ligation of CR2 bound to C3b with the B cell receptor-antigen complex lowers the B cell activation threshold by several orders of magnitude and is required for appropriate B cell activation *in vivo* [43]. Alternatively, non-cognate (antigen-nonspecific) B cells within the lymph nodes and spleens of mice have been shown to play a critical role in transporting IgM-containing ICs to follicular dendritic cells. This process is complement and complement receptor dependent and is essential for efficient germinal center formation and antibody affinity maturation [26, 44]. Non-cognate B cells with CR2 bound to ICs containing antigen can also present these ICs to antigen-specific T cells to induce appropriate T cell activation *in vitro* [45]. As such, molecules secreted by *S*. *aureus* that inhibit the association of ICs containing C3d with CR2 have the potential to influence the host adaptive immune response against this pathogen as suggested by others [42].

Taken together, our data provide evidence that *S*. *aureus* evades complement-mediated association with human B cells via expression of one or more virulence molecules regulated by SaeR/S. Although complement-mediated interaction of B cells with antigen contributes to the efficient generation of high affinity antibodies, it has long been known that all healthy adult humans have antibody specific for *S*. *aureus*. Notably, there are many *S*. *aureus* secreted molecules (not present in *S*. *epidermidis*) that are antigenic, and therefore, humans have antibodies to many of these secreted molecules. Further studies are required to test whether the ability of *S*. *aureus* to circumvent association with B-cells contributes to the microbe’s success as a human pathogen.

## References

[1] DeLeo FR, Otto M, Kreiswirth BN, Chambers HF. Community-associated meticillin-resistant *Staphylococcus aureus*. Lancet. 2010; 375: 1557–68. 10.1016/S0140-6736(09)61999-1 20206987 PMC3511788

[2] Centers for Disease Control and Prevention, Active Bacterial Core Surveillance (ABCs) Report, Emerging Infections Program Network, Methicillin-resistant Staphylococcus aureus, 2014. Available: http://www.cdc.gov/abcs/reports-findings/survreports/mrsa14.pdf.

[3] Landrum ML, Neumann C, Cook C, Chukwuma U, Ellis MW, Hospenthal DR, et al Epidemiology of *Staphylococcus aureus* blood and skin and soft tissue infections in the US military health system, 2005–2010. JAMA. 2012; 308: 50–9. 10.1001/jama.2012.7139 . 22760291

[4] Nubel U, Roumagnac P, Feldkamp M, Song JH, Ko KS, Huang YC, et al Frequent emergence and limited geographic dispersal of methicillin-resistant *Staphylococcus aureus*. Proc Natl Acad Sci U S A. 2008; 105: 14130–5. 10.1073/pnas.0804178105 . 18772392 PMC2544590

[5] Greenberg L. Antimicrobial activity of dermatomucosal agents. J Pharm Sci. 1968; 57: 1546–52. 10.1002/jps.2600570917 . 4877189

[6] Fowler VG Jr., Proctor RA. Where does a *Staphylococcus aureus* vaccine stand? Clin Microbiol Infect. 2014; 20 Suppl 5: 66–75. 10.1111/1469-0691.12570 . 24476315 PMC4067250

[7] Cheung AL, Bayer AS, Zhang G, Gresham H, Xiong YQ. Regulation of virulence determinants in vitro and in vivo in *Staphylococcus aureus*. FEMS Immunol Med Microbiol. 2004; 40: 1–9. . 14734180 10.1016/S0928-8244(03)00309-2

[8] Thammavongsa V, Kim HK, Missiakas D, Schneewind O. Staphylococcal manipulation of host immune responses. Nat Rev Microbiol. 2015; 13: 529–43. 10.1038/nrmicro3521 . 26272408 PMC4625792

[9] Serruto D, Rappuoli R, Scarselli M, Gros P, van Strijp JA. Molecular mechanisms of complement evasion: learning from staphylococci and meningococci. Nat Rev Microbiol. 2010; 8: 393–9. 10.1038/nrmicro2366 . 20467445

[10] Garcia BL, Ramyar KX, Ricklin D, Lambris JD, Geisbrecht BV. Advances in understanding the structure, function, and mechanism of the SCIN and Efb families of Staphylococcal immune evasion proteins. Adv Exp Med Biol. 2012; 946: 113–33. 10.1007/978-1-4614-0106-3_7 . 21948365 PMC3422867

[11] Voyich JM, Vuong C, DeWald M, Nygaard TK, Kocianova S, Griffith S, et al The SaeR/S gene regulatory system is essential for innate immune evasion by *Staphylococcus aureus*. J Infect Dis. 2009; 199: 1698–706. 10.1086/598967 . 19374556 PMC2799113

[12] Cheung GY, Wang R, Khan BA, Sturdevant DE, Otto M. Role of the accessory gene regulator *agr* in community-associated methicillin-resistant *Staphylococcus aureus* pathogenesis. Infect Immun. 2011; 79: 1927–35. 10.1128/IAI.00046-11 . 21402769 PMC3088142

[13] Falugi F, Kim HK, Missiakas DM, Schneewind O. Role of protein A in the evasion of host adaptive immune responses by *Staphylococcus aureus*. MBio. 2013; 4: e00575–13. 10.1128/mBio.00575-13 . 23982075 PMC3760252

[14] McDougal LK, Steward CD, Killgore GE, Chaitram JM, McAllister SK, Tenover FC. Pulsed-field gel electrophoresis typing of oxacillin-resistant *Staphylococcus aureus* isolates from the United States: establishing a national database. J Clin Microbiol. 2003; 41: 5113–20. 10.1128/JCM.41.11.5113-5120.2003 . 14605147 PMC262524

[15] Holden MT, Feil EJ, Lindsay JA, Peacock SJ, Day NP, Enright MC, et al Complete genomes of two clinical *Staphylococcus aureus* strains: evidence for the rapid evolution of virulence and drug resistance. Proc Natl Acad Sci U S A. 2004; 101: 9786–91. 10.1073/pnas.0402521101 . 15213324 PMC470752

[16] Voyich JM, Braughton KR, Sturdevant DE, Whitney AR, Said-Salim B, Porcella SF, et al Insights into mechanisms used by *Staphylococcus aureus* to avoid destruction by human neutrophils. J Immunol. 2005; 175: 3907–19. 10.4049/jimmunol.175.6.3907 . 16148137

[17] Nygaard TK, Pallister KB, Zurek OW, Voyich JM. The impact of alpha-toxin on host cell plasma membrane permeability and cytokine expression during human blood infection by CA-MRSA USA300. J Leukoc Biol. 2013; 94: 971–9. 10.1189/jlb.0213080 . 24026286 PMC3800068

[18] Lu T, Porter AR, Kennedy AD, Kobayashi SD, DeLeo FR. Phagocytosis and killing of *Staphylococcus aureus* by human neutrophils. J Innate Immun. 2014; 6: 639–49. 10.1159/000360478 . 24713863 PMC4140971

[19] Nygaard TK, Pallister KB, DuMont AL, DeWald M, Watkins RL, Pallister EQ, et al Alpha-toxin induces programmed cell death of human T cells, B cells, and monocytes during USA300 infection. PLoS One. 2012; 7: e36532 10.1371/journal.pone.0036532 . 22574180 PMC3344897

[20] Cheung GY, Rigby K, Wang R, Queck SY, Braughton KR, Whitney AR, et al *Staphylococcus epidermidis* strategies to avoid killing by human neutrophils. PLoS Pathog. 2010; 6: e1001133 10.1371/journal.ppat.1001133 . 20949069 PMC2951371

[21] Rogers DE, Tompsett R. The survival of staphylococci within human leukocytes. J Exp Med. 1952; 95: 209–30. 10.1084/jem.95.2.209 . 14907971 PMC2212057

[22] Parra D, Rieger AM, Li J, Zhang YA, Randall LM, Hunter CA, et al Pivotal advance: peritoneal cavity B-1 B cells have phagocytic and microbicidal capacities and present phagocytosed antigen to CD4+ T cells. J Leukoc Biol. 2012; 91: 525–36. 10.1189/jlb.0711372 . 22058420 PMC3317272

[23] Nakashima M, Kinoshita M, Nakashima H, Habu Y, Miyazaki H, Shono S, et al Pivotal advance: characterization of mouse liver phagocytic B cells in innate immunity. J Leukoc Biol. 2012; 91: 537–46. 10.1189/jlb.0411214 . 22058423

[24] Weis JJ, Tedder TF, Fearon DT. Identification of a 145,000 Mr membrane protein as the C3d receptor (CR2) of human B lymphocytes. Proc Natl Acad Sci U S A. 1984; 81: 881–5. 10.1073/pnas.81.3.881 . 6230668 PMC344942

[25] Boackle SA, Morris MA, Holers VM, Karp DR. Complement opsonization is required for presentation of immune complexes by resting peripheral blood B cells. J Immunol. 1998; 161: 6537–43. 10.1016/S0161-5890(98)90633-2 . 9862679

[26] Phan TG, Grigorova I, Okada T, Cyster JG. Subcapsular encounter and complement-dependent transport of immune complexes by lymph node B cells. Nat Immunol. 2007; 8: 992–1000. 10.1038/ni1494 . 17660822

[27] Link A, Zabel F, Schnetzler Y, Titz A, Brombacher F, Bachmann MF. Innate immunity mediates follicular transport of particulate but not soluble protein antigen. J Immunol. 2012; 188: 3724–33. 10.4049/jimmunol.1103312 . 22427639

[28] Fitzgerald JR, Sturdevant DE, Mackie SM, Gill SR, Musser JM. Evolutionary genomics of *Staphylococcus aureus*: insights into the origin of methicillin-resistant strains and the toxic shock syndrome epidemic. Proc Natl Acad Sci U S A. 2001; 98: 8821–6. 10.1073/pnas.161098098 . 11447287 PMC37519

[29] Malachowa N, Whitney AR, Kobayashi SD, Sturdevant DE, Kennedy AD, Braughton KR, et al Global changes in *Staphylococcus aureus* gene expression in human blood. PLoS One. 2011; 6: e18617 10.1371/journal.pone.0018617 . 21525981 PMC3078114

[30] van den Elsen JM, Isenman DE. A crystal structure of the complex between human complement receptor 2 and its ligand C3d. Science. 2011; 332: 608–11. 10.1126/science.1201954 . 21527715

[31] Fearon DT. Human complement receptors for C3b (CR1) and C3d (CR2). J Invest Dermatol. 1985; 85: 53s–7s. 10.1111/1523-1747.ep12275473 . 2989379

[32] Dossett JH, Kronvall G, Williams RC Jr., Quie PG. Antiphagocytic effects of staphylococfcal protein A. J Immunol. 1969; 103: 1405–10. . 4188886

[33] Sjodahl J. Repetitive sequences in protein A from *Staphylococcus aureus*. Arrangement of five regions within the protein, four being highly homologous and Fc-binding. Eur J Biochem. 1977; 73: 343–51. 10.1111/j.1432-1033.1977.tb11324.x . 557409

[34] Cary S, Krishnan M, Marion TN, Silverman GJ. The murine clan V(H) III related 7183, J606 and S107 and DNA4 families commonly encode for binding to a bacterial B cell superantigen. Mol Immunol. 1999; 36: 769–76. 10.1016/S0161-5890(99)00085-1 . 10593515

[35] Goodyear CS, Silverman GJ. Death by a B cell superantigen: In vivo VH-targeted apoptotic supraclonal B cell deletion by a staphylococcal toxin. J Exp Med. 2003; 197: 1125–39. 10.1084/jem.20020552 . 12719481 PMC2193973

[36] Montgomery CP, Boyle-Vavra S, Daum RS. Importance of the global regulators Agr and SaeRS in the pathogenesis of CA-MRSA USA300 infection. PLoS One. 2010; 5: e15177 10.1371/journal.pone.0015177 . 21151999 PMC2996312

[37] Nygaard TK, Pallister KB, Ruzevich P, Griffith S, Vuong C, Voyich JM. SaeR binds a consensus sequence within virulence gene promoters to advance USA300 pathogenesis. J Infect Dis. 2010; 201: 241–54. 10.1086/649570 . 20001858 PMC2798008

[38] Woehl JL, Stapels DA, Garcia BL, Ramyar KX, Keightley A, Ruyken M, et al The extracellular adherence protein from *Staphylococcus aureus* inhibits the classical and lectin pathways of complement by blocking formation of the C3 proconvertase. J Immunol. 2014; 193: 6161–71. 10.4049/jimmunol.1401600 . 25381436 PMC4258549

[39] Rooijakkers SH, Ruyken M, Roos A, Daha MR, Presanis JS, Sim RB, et al Immune evasion by a staphylococcal complement inhibitor that acts on C3 convertases. Nat Immunol. 2005; 6: 920–7. 10.1038/ni1235 . 16086019

[40] Koch TK, Reuter M, Barthel D, Bohm S, van den Elsen J, Kraiczy P, et al *Staphylococcus aureus* proteins Sbi and Efb recruit human plasmin to degrade complement C3 and C3b. PLoS One. 2012; 7: e47638 10.1371/journal.pone.0047638 . 23071827 PMC3469469

[41] Burman JD, Leung E, Atkins KL, O'Seaghdha MN, Lango L, Bernado P, et al Interaction of human complement with Sbi, a staphylococcal immunoglobulin-binding protein: indications of a novel mechanism of complement evasion by *Staphylococcus aureus*. J Biol Chem. 2008; 283: 17579–93. 10.1074/jbc.M800265200 . 18434316 PMC2649420

[42] Ricklin D, Ricklin-Lichtsteiner SK, Markiewski MM, Geisbrecht BV, Lambris JD. Cutting edge: members of the *Staphylococcus aureus* extracellular fibrinogen-binding protein family inhibit the interaction of C3d with complement receptor 2. J Immunol. 2008; 181: 7463–7. Epub 2008/11/20. 10.4049/jimmunol.181.11.7463 . 19017934 PMC2673544

[43] Tedder TF, Inaoki M, Sato S. The CD19-CD21 complex regulates signal transduction thresholds governing humoral immunity and autoimmunity. Immunity. 1997; 6: 107–18. 10.1016/S1074-7613(00)80418-5 . 9047233

[44] Phan TG, Green JA, Gray EE, Xu Y, Cyster JG. Immune complex relay by subcapsular sinus macrophages and noncognate B cells drives antibody affinity maturation. Nat Immunol. 2009; 10: 786–93. 10.1038/ni.1745 . 19503106 PMC2776777

[45] Thornton BP, Vetvicka V, Ross GD. Natural antibody and complement-mediated antigen processing and presentation by B lymphocytes. J Immunol. 1994; 152: 1727–37. . 8120381

